# PtNPs/PEDOT:PSS-Modified Microelectrode Arrays Reveal Electrophysiological Activities of Different Neurons in Medial Amygdala of Mice Under Innate Fear

**DOI:** 10.3389/fnins.2022.868235

**Published:** 2022-05-10

**Authors:** Penghui Fan, Yilin Song, Botao Lu, Yiding Wang, Yuchuan Dai, Jingyu Xie, Enhui He, Zhaojie Xu, Gucheng Yang, Fan Mo, Juntao Liu, Mixia Wang, Xinxia Cai

**Affiliations:** ^1^State Key Laboratory of Transducer Technology, Aerospace Information Research Institute, Chinese Academy of Sciences, Beijing, China; ^2^School of Electronic, Electrical and Communication Engineering, University of the Chinese Academy of Sciences, Beijing, China

**Keywords:** conductive polymer, MEA, fear processing, electrophysiology, medial amygdala, freely behaving

## Abstract

The medial amygdala (MA) plays an important role in the innate fear circuit. However, the electrophysiological mechanism of MA for processing innate fear needs to be further explored. In this study, we fabricated microelectrode arrays (MEAs) with detecting sites arranged to match the location and shape of MA in mice and detected the electrophysiology in freely behaving mice under 2-methyl-2-thiazoline (2MT)-induced fear. The detection performance of MEA is improved by modifying metal nanoparticles and conductive polymers (PtNPs/PEDOT:PSS). After modification, the impedance magnitude and phase of electrodes were decreased to 27.0 ± 2.3 kΩ and −12.30 ± 0.52°, respectively, leading to a signal-to-noise ratio of 10. Its electrochemical stability and mechanical stability were also verified by cyclic voltammetry (CV) sweeping and ultrasonic vibration. MEAs were then implanted into the MA of mice, and the electrophysiology and behavioral characteristics were synchronously recorded and analyzed. The results showed that 2MT induced strong defensive behaviors in mice, accompanied by increases in the average spike firing rate and local field potential (LFP) power of MA neurons. According to principles commonly applied to cortical extracellular recordings, the recorded neurons are divided into two classes based on waveforms. Statistics showed that about 37% of type 1 neurons (putative GABAergic neurons) and 87% of type 2 neurons (putative glutamatergic neurons) were significantly activated under innate fear. At the same time, the firing rate of some activated neurons had a good linear correlation with the freezing rate.

## Introduction

Fear is a natural and primitive emotion in humans and animals, which can alert themselves in the presence of physical or psychological dangers (Gross and Canteras, 2012). Responses under fear involve behavioral, biochemical, and emotional reactions, such as defensive behaviors, increased adrenaline levels, and being more excited. Sometimes, fear is also a symptom of some mental diseases, such as phobia, posttraumatic stress disorder, and obsessive-compulsive disorder (Kessler et al., 2005; McLean et al., 2011; Dias et al., 2013; Gregory et al., 2020). The study of the neural circuits processing the fear can help us better understand the pathogenic mechanisms of related diseases, so neuroscientists are committed to researching and addressing this issue. Fear is theoretically classified into learned fear and innate fear (Duvarci and Pare, 2014). Innate fear can be naturally triggered by aversive stimuli such as electric shock to the foot, lack of ambient oxygen, and predator odor, which does not depend on the learning process establishing an association between responses and specific stimuli (Silva et al., 2016).

For rodent, olfaction is the most important sensory system to avoid danger by detecting different odors. Innate fear in rodent has been reported with several predator odors, such as cat fur, fox urine, 2-methyl-2-thiazoline (2MT), and 2,4,5-dihydro-2,5 trimethylthiazoline (TMT), a synthesized component of fox anal secretions. Previous studies have shown that predator odor-induced fear is related to multiple brain regions. A major signal pathway is that these odor signals are detected by the vomeronasal organ (VNO) located at the bottom of the nasal cavity and then transmitted to the medial amygdala (MA) through the auxiliary olfactory bulb (AOB). This information is then processed and integrated in the hypothalamus and finally arrives in the PAG to control the occurrence of defensive response behaviors (Silva et al., 2016). MA connects the detection unit and the integration unit, which is very important in the whole circuit. MA encoding and regulating aggression, social interaction, and sexual behavior has been extensively studied by electrophysiological recording, optogenetics, chemogenetics, *in vivo* microendoscopic Ca^2+^ imaging and so on (Hong et al., 2014; Unger et al., 2015; Li et al., 2017; Haller, 2018; Chen et al., 2019). However, in the study of the mechanism of MA under innate fear, researchers currently mainly used c-fos mapping, lesion, drug regulation, electrophysiological detection under anesthesia, and other technical means. For example, Isosaka et al. demonstrated that the neurons in MA were activated after fear by c-fos staining technique, but they did not further study the types and proportion of activated neurons (Isosaka et al., 2015). In addition, Li et al. found that MA lesions reduced freezing and increased the frequency of contact with predator odor sources (Li et al., 2004; Blanchard et al., 2005; Takahashi et al., 2007). Furthermore, studies have shown that the temporary inactivation of MA by local injection of GABA receptor agonist muscimol leads to the complete blocking of TMT-induced freezing (Müller and Fendt, 2006). Several previous electrophysiological studies have proved that predator odor exposure can activate partial MA neurons (Bergan et al., 2014; Govic and Paolini, 2015). However, previous electrophysiological studies were carried out under anesthesia, and the studies focused only on the brief response to aversive odor exposure for several seconds. *In vivo* electrophysiological recording conduces to revealing the neural mechanisms of the innate fear processing, which includes measuring the action potentials of a single neuron (spikes) and population cell neural activities (Belitski et al., 2008; Buzsáki et al., 2012; Spira and Hai, 2013). Therefore, it is necessary and urgent to explore the neural mechanism of freely behaving mice under the persistent fear state by electrophysiological techniques.

The microelectrode arrays (MEAs) are considered as the ideal platform for electrophysiological detection. Compared with traditional microwire electrodes, its detection site arrangement is more controllable. Nanomaterials have been widely used in the surface modification on the electrode detection sites to improve the detection sensitivity and signal-to-noise ratio. Platinum black nanoparticles (PtNPs) can effectively reduce the electrode impedance because of their high specific surface area (Desai, 2010; Zhang et al., 2016; Xie et al., 2020). Poly(3,4-ethylene-dioxythiophene) (PEDOT) is a popular class of conductive polymers due to its chemical stability and relatively high conductivity (Gerwig et al., 2012; Panigrahy and Kandasubramanian, 2020; Cogal, 2021). In addition, it can support charge injection capacities of ≈15 mC/cm^2^, which is roughly three times higher than that of iridium oxide (Cogan, 2008). However, as a polymer coating, the interaction between PEDOT and the underlying noble metal electrode is often very weak (Luo et al., 2011; Snook et al., 2011; Boehler et al., 2017). So adding a layer of metal nanoparticles between PEDOT layer and metal electrode may make PEDOT more firmly adhere to the electrode and further reduce the electrode impedance.

In this study, we started with more advanced electrophysiological sensors and analysis methods to explore the neural mechanism of MA under innate fear. In terms of electrophysiological sensors, we designed and manufactured MEAs for the long-term detection of neural activities in the MA of mice. The impedance and phase delay of electrodes modified with PtNPs/PEDOT:PSS were significantly decreased, and the signal-to-noise ratio can reach 10. At the same time, the electrode also has excellent electrochemical stability and mechanical stability. In terms of analysis methods, we divided the whole analysis process into three stages, namely, control, avoidance, and freezing, with a total of 12 min. The neural mechanism of MA under 2MT-induced innate fear was explored by synchronously recording electrophysiological activities and behavioral characteristics of mice.

## Materials and Methods

### Reagents and Apparatus

The chloroplatinic acid (H_2_PtCl_6_) and lead acetate [Pb (CH_3_COO)_2_] were purchased from Sinopharm Chemical Reagent (China). 3,4-ethoxylenedioxythiophene (EDOT) was purchased from Aladdin (China). Poly(sodium-4-styrenesulfonate) (PSS) was obtained from Herochem (China). 2-methyl-2-thiazoline (2MT) was purchased from Shanghai Acmec Biochemical Co., Ltd (China). 1,1′-Dioctadecyl-3,3,3′,3′-tetramethylindocarbocyanine perchlorate (DiI) was purchased from Beyotime Institute of Biotechnology (China). The stereotaxic frame (51600) was purchased from Stoelting (USA). Micropositioner (model 2662) was purchased from David KOPF instrument (USA).

### Subjects

Six female mice (C57BL/6J) weighing 20–25 g were used in the experiment, which were purchased from Charles River Laboratory Animal Technology Co, Ltd. All mice were housed at 20°C with a 12-h light: 12-h dark reversed light cycle. All animal experiments were conducted with the permission of the Beijing Association on Laboratory Animal Care and approved by the Institutional Animal Care and Use Committee at Aerospace Information Research Institute, Chinese Academy of Science (AIRCAS).

### Microelectrode Arrays' Design, Manufacture, and Modification

The MEAs, based on micro-electro-mechanical system (MEMS) technology, were developed for electrophysiological recording in MA of freely behaving mice. The MA area is similar to an irregular triangle and is located at a depth of about 5 mm in the brain (Supplementary Figure S1A). The widest part of MA is only about 500 μm, and the longitudinal length is about 1,000 μm. In order to match the shape and size of the MA, there were eight electrode recording sites (15 μm in diameter) on the tip of every shank forming a 2 × 8 array (Supplementary Figure S1B). The MEAs were designed to consist of two 7-mm-long shanks. Each shank has a width of 180 μm, a thickness of 30 μm, and a shank-to-shank distance of 80 μm.

**Figure 1:**
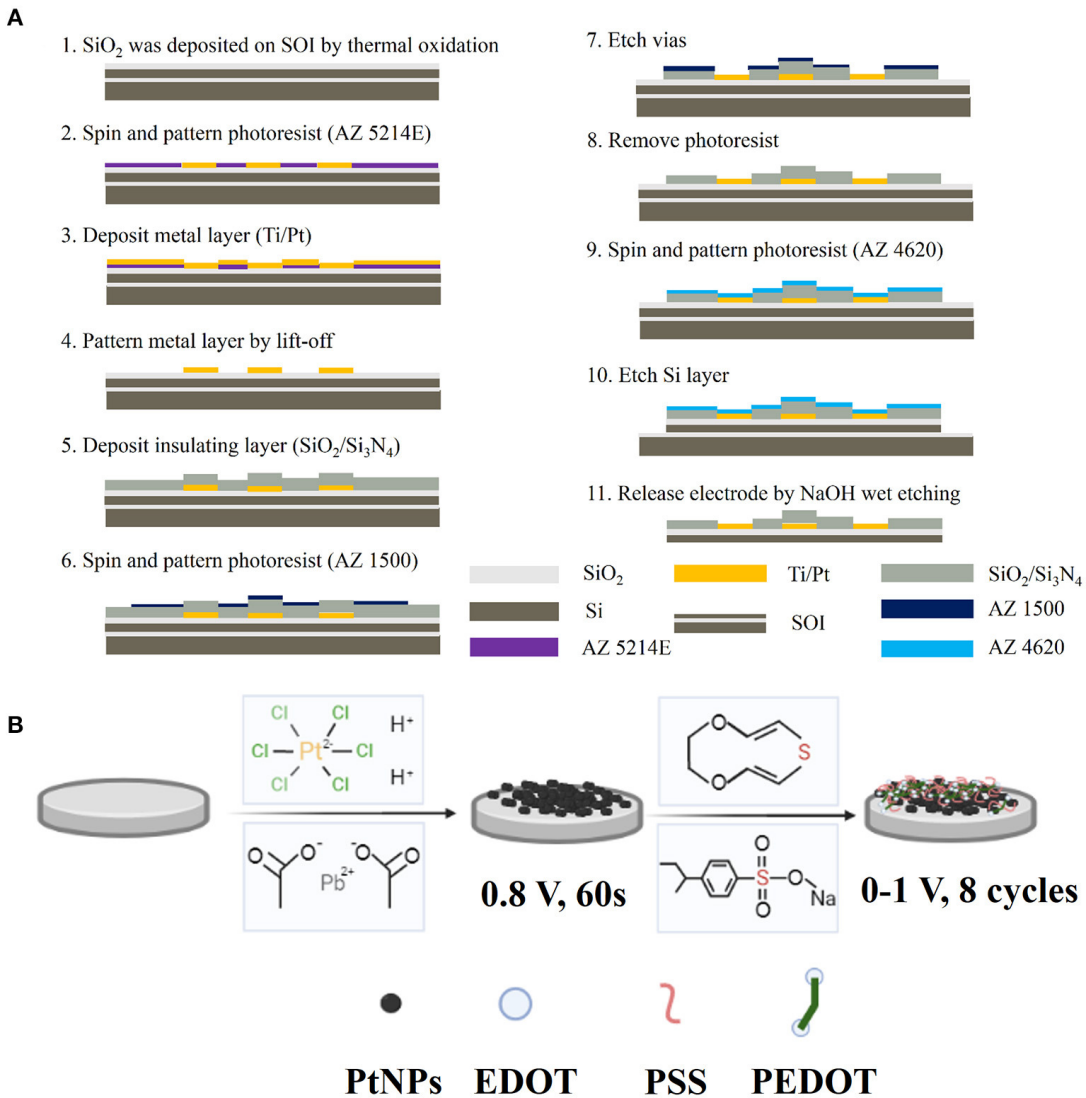
MEA fabrication and modification diagram. **(A)** Manufacturing process flow of MEA; **(B)** Schematic diagram of PtNPs/PEDOT:PSS modification method.

The MEA consists of three layers, namely, base layer Si (30 μm), metal layer Ti/Pt (30 nm/250 nm), and insulating layer SiO_2_/Si_3_N_4_ (300 nm/500 nm) (Supplementary Figure S1B). The electrode manufacturing process flow is shown in Figure 1A. The fabrication process can be briefly divided into three parts: First, the Ti/Pt conductive layer pattern (including recording sites, bonding pads, and conducting wire) was formed by photolithography, sputtering, and lift-off. Then, SiO_2_/Si_3_N_4_ insulating layer was deposited, and the recording sites and bonding pads were exposed by the second photolithography and CHF_3_ reactive ion etching (RIE). Finally, each electrode is released by KOH wet etching.

To decrease the electrode impedance and improve the signal-to-noise ratio, PtNPs and PEDOT:PSS were successively electroplated onto the bare microelectrodes. The schematic diagram of PtNPs/PEDOT:PSS electrode modification is shown in Figure 1B. Chloroplatinic acid (48 mM in PBS) and lead acetate (4.2 mM in PBS) are mixed at a volume ratio of 1:1 and kept for 12 h to obtain a platinum (Pt) black electrolyte solution. EDOT solution (20 mM) was obtained by adding 0.0284 g of EDOT (20 mM) and 0.206 g of PSS (0.1 M) to 10 ml of deionized water and treating with ultrasound for 1 h. Electrode surface modification was completed by an electrochemical workstation (Gamry Reference 600, Gamry Instruments, USA). First, Pt black nanoparticles were electroplated by chronoamperometry (-0.8 V, 50 s) using a Pt wire as the counter electrode and then PEDOT by cyclic voltammetry (CV) (0–1 V, 8 cycle) using an Ag/AgCl as reference electrode and a Pt wire as the counter electrode.

### *In vitro* Characterization

Microscope and scanning electron microscope (SEM) were used to observe the surface morphology before and after electrode modification. Both CV and electrochemical impedance spectroscopy (EIS) were carried out in phosphate-buffered saline solution (PBS, 0.1M, PH = 7.4) with an Ag/AgCl as the reference electrode and a Pt wire as the counter electrode. Charge storage capacity (CSC) was measured by CV from −0.6 V to 0.8 V at a scanning rate of 0.1 V/s. Electrode stability was tested by CV sweeping from −0.6 V to 0.8 V (6,000 cycle) and ultrasonic oscillation (50 W, 100 min) in an ultrasonic cleaner.

### Surgery

The operating table and all surgical instruments are cleaned and disinfected in advance. The mice were anesthetized by the isoflurane (3% for induction and 1% for maintenance) and then fixed in the stereotaxic frame. Then, the mice scalp was cut off to expose the skull, and the head was adjusted to a level: one craniotomy for implanting MEA to MA (AP: −1.5 mm, ML: 2.0 mm) and the other three for skull nail as reference electrodes. The MEAs were advanced to a depth of 5.2 mm from the cortical surface at a speed of 5 μm/s using a micropositioner. Finally, the MEAs were fixed by dental cement, and the mice were put on the heating pad until woke up.

After the experiment, the mouse was injected with 10% chloral hydrate into deep anesthesia and perfused through the heart with 0.9% NaCl and 4% paraformaldehyde in sequence. The brain was removed and put into 20% sucrose solution for 24 h followed by 30% sucrose solution for 48 h for postfixation. Then, 40 μm thick sections were prepared with a freezing microtome and were observed under a microscope. According to the mouse brain atlas (Paxinos and Franklin, 2001), the electrode implantation position is mainly concentrated in MePD (posterodorsal part of MA). The schematic diagram of electrode positions of six experimental mice is shown in Supplementary Figure S2.

### Experimental Strategy

**Figure 2:**
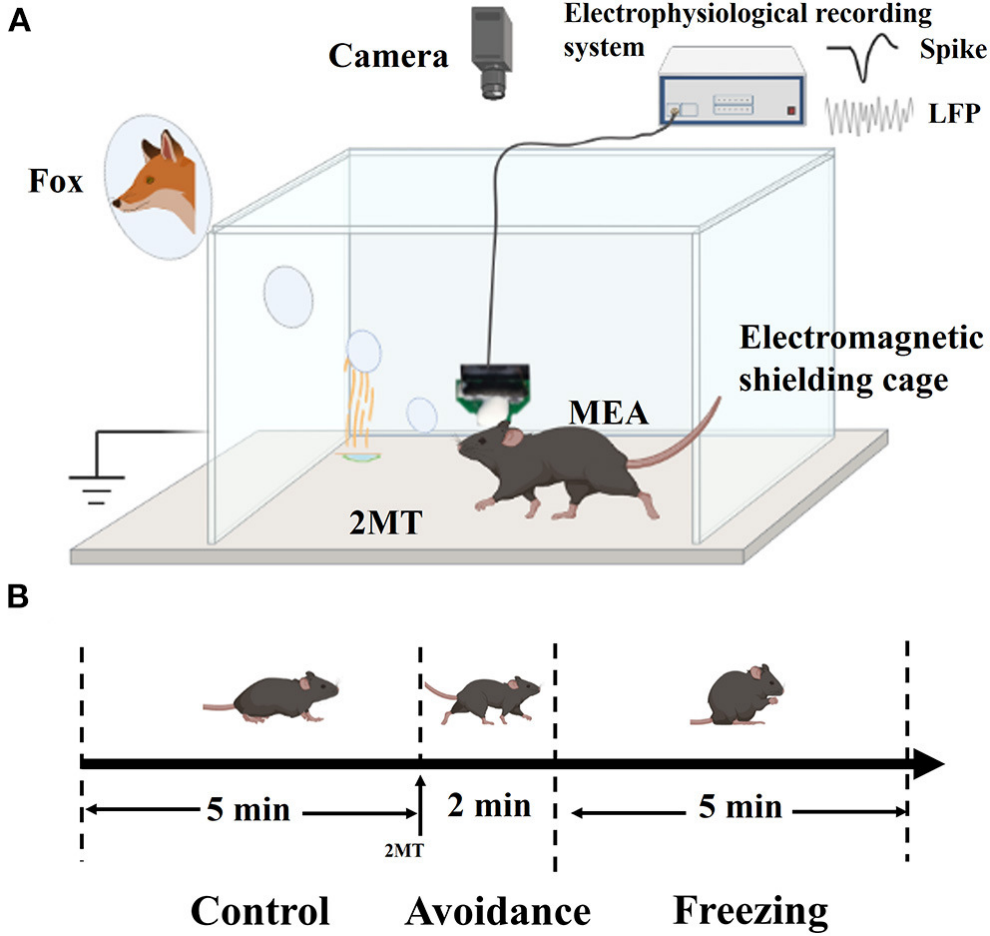
Schematic diagram of experiment platform and experimental flow. **(A)** Schematic diagram of experiment platform; **(B)** Three behavioral stages in the experimental process.

As shown in Figure 2A, the mice with MEAs were placed in a 20 × 20 × 16 cm (L × W × H) shielded box with a 2 × 2 cm paper on the bottom. The electrophysiological signals in MA were recorded in real time, and the behavior changes of mice were recorded simultaneously by a camera (Hikvision, China). The recording video frame rate is 25 Hz. Before the experiment, the mice were allowed to habit in the box for 15 min until they were free to go anywhere in the box. First, electrophysiological signals and behavior video were recorded for 5 min as a control group. Then, 5 μl of 2MT (25%) was added onto the bottom paper through the pipette, and the data were continuously recorded for 10 min.

In the experiment, we observed that the mice would exhibit thigmotaxis (a special behavior that prefers to be close to the wall) and even jump after dropping 2MT. This behavior would last about 2 min, and it is defined as the avoidance stage. Then, the mice exhibited strong freezing, and it is defined as the freezing stage. In order to facilitate statistics and analysis, we divided the entire behavior stage into the control stage, avoidance stage, and freezing stage, with durations of 5 min, 2 min, and 5 min, respectively (Figure 2B). In order to analyze the synchronous changes in mouse behavior and electrophysiological signals, the freezing and spike firing rate are computed in 30-s bins.

### Data Analysis

All data are expressed as mean ± SE. Significance analysis uses the one-way ANOVA or one-way repeated measures ANOVA test by Origin. The analysis of behavioral characteristics is completed with the help of behavioral analysis software EthoVision. Among them, freezing is defined as a state in which there is no movement except breathing-related movement and lasts for more than 1.5 s (Müller and Fendt, 2006). The time of freezing (in seconds) and the velocity (in centimeters/second) were collected with EthoVision. The freezing rate in this study is the percentage of freezing time to the total time.

Electrophysiological signals are obtained by plugging MEAs into the homemade electrophysiological recording system (Xu et al., 2018; He et al., 2021). The signal sampling rate is 30 K Hz. A high pass filter was applied at 250 Hz to obtain spikes, and a low pass filter was applied at 250 Hz to view the local field potential (LFP). The software written by our laboratory was used for spike sorting/clustering. Units were isolated based on waveform parameters including peak amplitude and principal components. Clusters containing similar valid waveforms were manually defined, and the single units did not contain a refractory period <1 ms. MANOVA test (*p* < 0.05) was used to validate the single-unit nature of spikes (Friend et al., 2015). Neuroexplorer (Plexon) was used for further analysis.

## Results

### Characterization of PtNPs/PEDOT:PSS-Modified MEA

**Figure 3:**
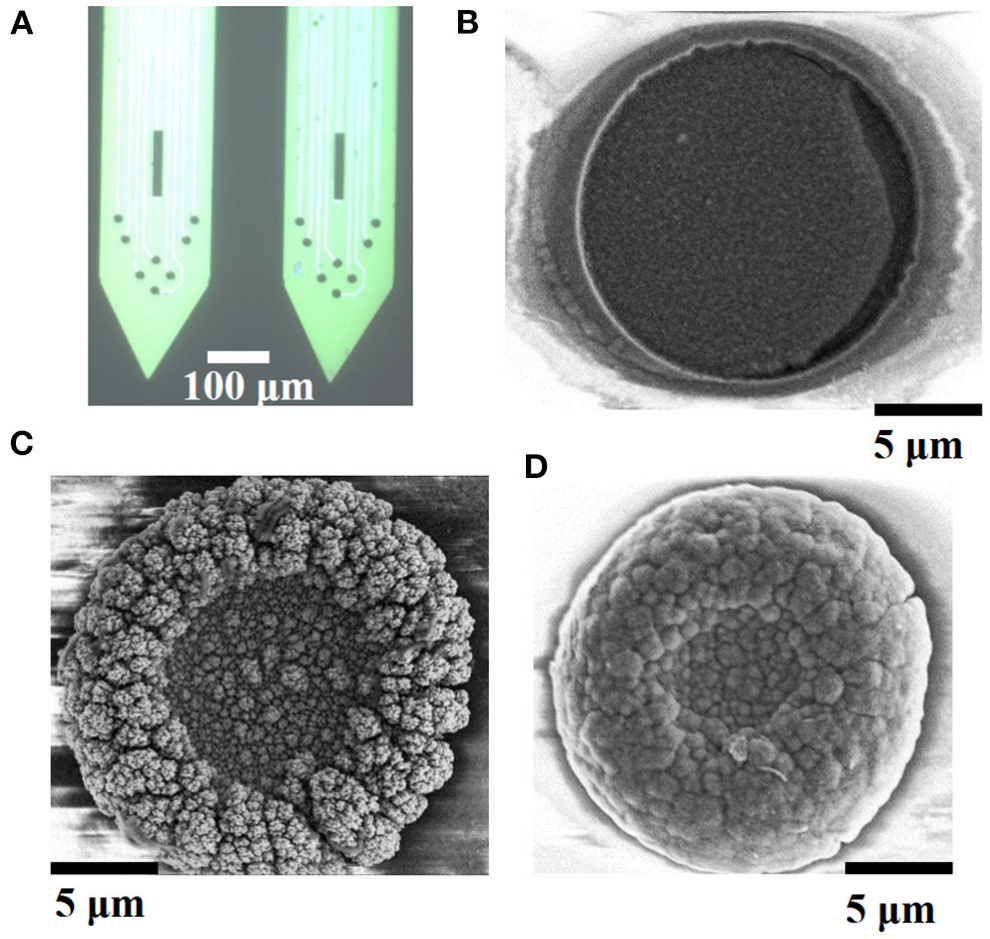
Electrode modification topography. **(A)** Microscopic image of modified PtNPs/PEDOT:PSS electrode. The morphology of **(B)** PEDOT:PSS, **(C)** PtNPs, and **(D)** PtNPs/PEDOT:PSS modification schemes under scanning electron microscope.

The detection performance of bare electrode is poor due to its large impedance. PtNPs modification is beneficial to reduce the impedance of the electrode and obtain a better signal-to-noise ratio. PEDOT modification can improve the biocompatibility and long-term signal detection ability. In this study, we first electroplated PtNPs on the electrode and then electroplated PEDOT:PSS on the PtNPs-modified electrode. The PtNPs/PEDOT:PSS-modified electrode is shown in Figure 3A. In order to compare the electrode performance of different modification methods, PEDOT:PSS, PtNPs, and PtNPs/PEDOT:PSS were plated on the bare electrode according to the method described earlier, and the morphology of modified electrode sites was characterized using a SEM, as shown in Figures 3B–D. The morphology of PEDOT:PSS modification is relatively flat. However, the specific surface area of the electrode did not increase significantly. The PtNPs-modified electrode formed an obvious particle bulge on the surface, increasing the specific surface area of the electrode. The better biocompatibility of PEDOT has been confirmed by many studies (Kozai et al., 2015; Pranti et al., 2018; Dijk et al., 2020). In order to ensure lower impedance and better biocompatibility, we electroplated PEDOT:PSS on PtNPs-modified electrode. The morphology of PtNPs/PEDOT:PSS-modified electrode is shown in Figure 3D. PEDOT fills the gap formed by PtNPS, like covering the surface of PtNPs with a thin film to make the electrode surface smoother.

**Figure 4:**
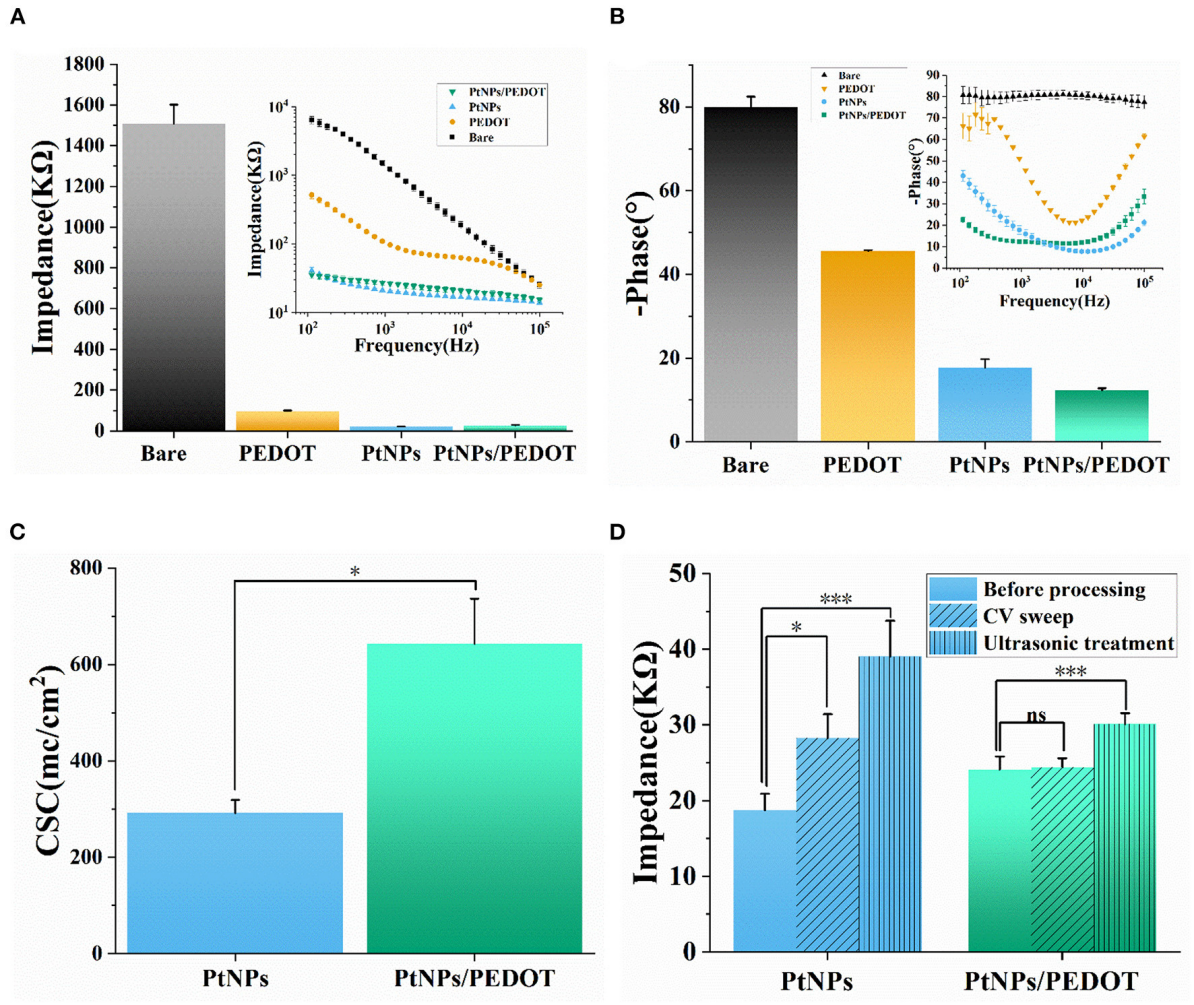
Electrode characterization and stability test. **(A,B)** Average impedance and phase delay of the different modified electrodes at 1 kHz (*n* = 5 recording sites, the illustration shows the phase delay and impedance at 100 Hz−100 kHz scanning frequency). **(C)** CSC of PtNPs/PEDOT:PSS- and PtNPs-modified electrode (*n* = 4 recording sites). **(D)** Impedance (1 kHz) of PtNPs and PtNPs/PEDOT:PSS electrodes after CV scanning and ultrasonic vibration (*n* = 4 recording sites). Data are means ± SE. One-way ANOVA for statistical analysis in **(A–C)**, and one-way repeated measures ANOVA for statistical analysis in **(D)**. See Supplementary Material Tables 1, 4 for statistical analysis in **(A)** and **(B)**. *p < 0.05; ***p < 0.001; ns, *p* > 0.05.

As shown in Figure 4A, the average impedance of PtNPs/PEDOT:PSS-modified electrode is 27.0 ± 2.3 KΩ, which decreased by more than 90% compared with the bare electrode and was also only a quarter of the PEDOT-modified electrode. In terms of phase delay, the average phase delay of PtNPs/PEDOT:PSS-modified electrode at 1 kHz is about −12.30 ± 0.52° (Figure 4B), which is lower than that of PtNPs-modified electrode (-17.77 ± 2.00°). The average phase delay of PtNPs and PtNPs/PEDOT:PSS-modified electrodes at useful bandwidth for recording spikes (1–3 kHz) is shown in Supplementary Figure S3. CSC is another common indicator for electrode material characterization (Yamaguchi et al., 2016; Davidsen et al., 2019). We compared the CSC of the PtNPs and PtNPs/PEDOT:PSS-modified electrode. The results show that the CSC of PtNPs/PEDOT:PSS-modified electrode is almost twice that of PtNPs, which shows the good performance of PtNPs/PEDOT:PSS modification (Figure 4C). The electrode durability is particularly important for the electrophysiological measurement of freely behaving mice. In order to test the electrochemical and mechanical stability of the electrode, we performed the CV sweeping and ultrasonic oscillation on the electrodes (Figure 4D). After 6,000 cycles of CV sweeping, the impedance of PtNPs-modified electrodes increased significantly, but the impedance of PtNPs/PEDOT:PSS-modified electrodes hardly changed. In addition, the average impedance of PtNPs/PEDOT:PSS-modified electrodes was already less than that of PtNPs-modified electrodes. At the same time, the average phase delay of PtNPs-modified electrode increased to −50°, while PtNPs/PEDOT:PSS-modified electrode could still be maintained within −30° (Supplementary Figure S4). After 100 min ultrasonic oscillation, the impedance and phase delay of PtNPs-modified electrode increased further, while the phase delay of PtNPs/PEDOT:PSS electrode did not change significantly in this process. This proved the good stability of PtNPs/PEDOT:PSS modification.

In addition, we tested the background noise of PtNPs electrode and PtNPs/PEDOT:PSS electrode in PBS. The results show that PtNPs/PEDOT:PSS electrode has less background noise (Supplementary Figure S5A,B). In the electrophysiological test experiment, the PtNPs/PEDOT:PSS-modified electrode shows the characteristics of low noise and high signal-to-noise ratio. As shown in Supplementary Figure S5C, the noise of the signal measured by the modified electrode is about ± 15 μV, and the signal-to-noise ratio can reach about 10.

### Electrophysiology and Behavioral Characteristics of Mice Under Fear

**Figure 5:**
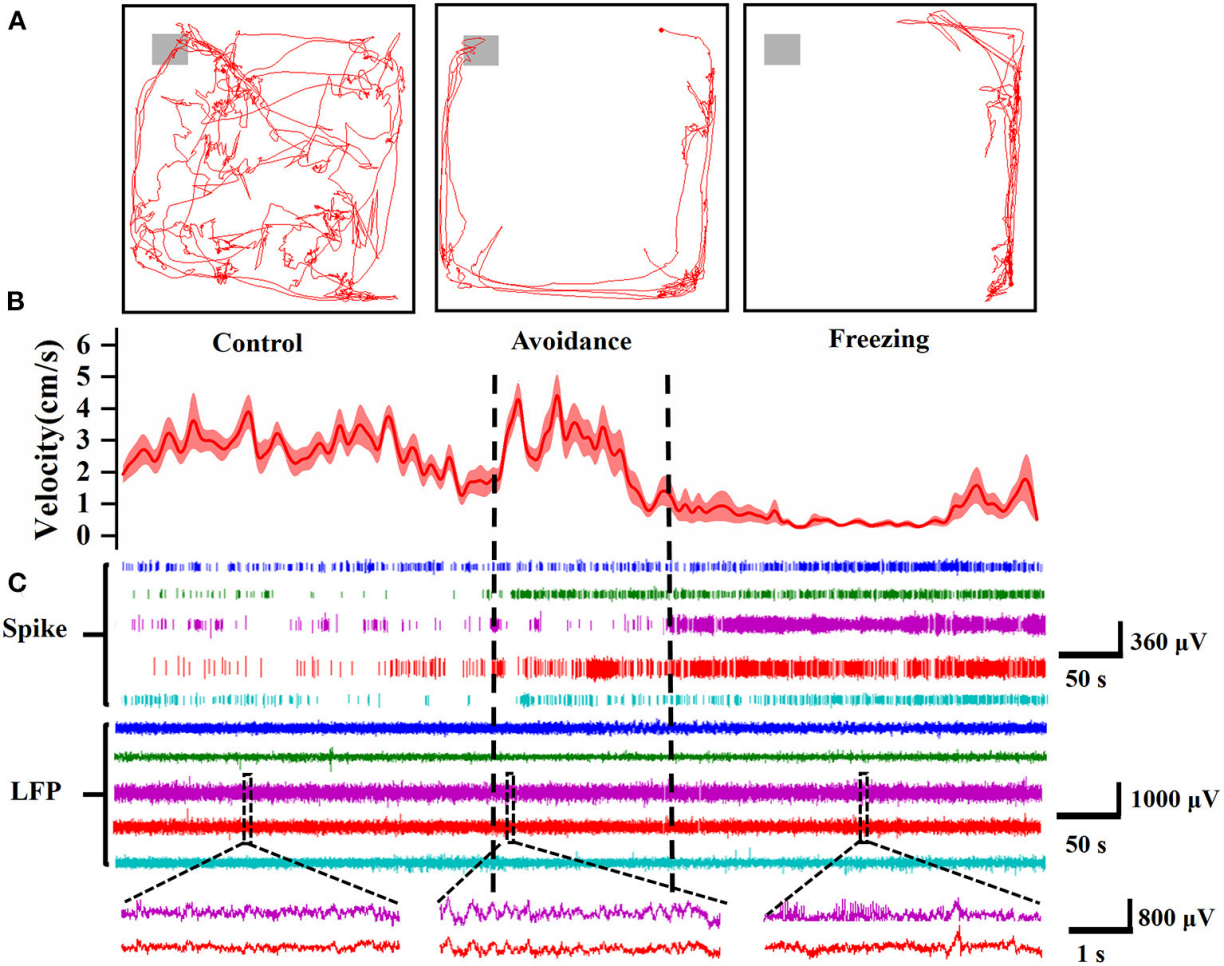
Electrophysiological and behavioral characteristics of mice in three stages. **(A)** The trajectory diagram of the mouse in the control stage (left, 5 min), avoidance stage (middle, 2 min), and freezing stage (right, 5 min), and the gray area represents the range of the 2MT paper. **(B)** The velocity of the mice in three stages (*n* = 6 mice). **(C)** Spike and LFP of 5 typical channels in different mice. At the bottom is an enlarged view of the two channels corresponding to the dotted line in the LFP (5 s).

We recorded the electrophysiology of MA and behavior of mice simultaneously. In terms of behavior, the mice freely explore anywhere in the box in the control stage. After adding 2MT, the mice will walk along the edge of the box most of the time and exhibit thigmotaxis, an index of increased anxiety (Kennedy et al., 2020). In the avoidance stage, the mice will want to approach and explore the odor source, but they are more likely to walk rapidly along the wall of the behavior box or freeze, and even individual mice exhibit jump. The mice's movement velocity increased and decreased sharply, indicating that the mice were panicked (Figures 5A,B). After entering the freezing stage, the mice curled up in a corner and showed strong freezing defense behavior. The velocity of mice also reached the lowest (refer to Supplementary Materials for video examples). In order to confirm the reliability of the results, we replaced 2MT with deionized water in the control experiment. The results showed that the dripping water had no effect on the behavior of mice, which indicated that the defense behavior of mice was induced by 2MT (Supplementary Figure S6).

In terms of electrophysiology, the spike firing rate increased significantly in the avoidance and freezing stages, which reflected that the neurons in MA were activated after fear (Figure 5C). The LFP also became more intense and different. It can be seen from the enlarged figure at the bottom that the LFP amplitude becomes larger in the avoidance stage. In the freezing stage, in addition to the larger amplitude compared with the control stage, there are more dense spines in the LFP waveform, which may be caused by the increase in action potential.

**Figure 6:**
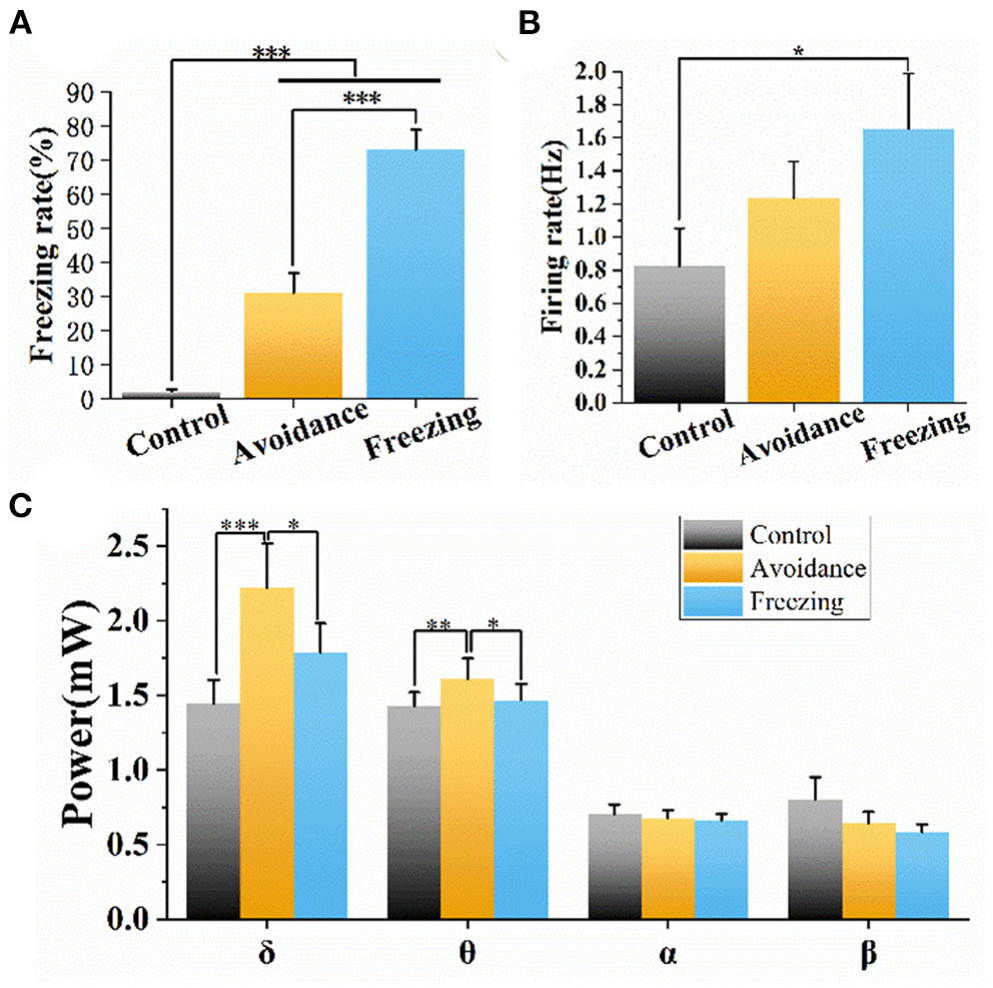
Freezing rate, firing rate, and LFP power of mice in the three stages. **(A)** Freezing rate of mice in the three stages. **(B)** Firing rate of mice in the three stages. **(C)** LFP power of different frequency bands in three stages. Data are means ± SE. One-way repeated measures ANOVA. *p < 0.05; **p < 0.01; ***p < 0.001. *n* = 6 mice.

We also counted the freezing rate, spike firing rate, and LFP power to quantitatively analyze the three stages of fear. It can be seen from Figure 6A that the average freezing rate of six mice is <10% in the control stage. Then, the freezing rate gradually increased in the avoidance and freezing stages, indicating that 2MT induced a strong defense response in mice. At the same time, we counted the action potential firing rate of 69 neurons in six mice. We found that the firing rate and the freezing rate had the same trend in the three stages, and the firing rate in the freezing stage was significantly different from that in the control stage (Figure 6B). The LFP power is mainly concentrated in the low-frequency band, and we counted the LFP power of four different frequency bands, namely, δ (1–4 Hz), θ (4–8 Hz), α (8–13 Hz), and β (13–30 Hz) (Figure 6C). We found that the LFP power also has a similar trend, that is, the δ and θ band powers in the avoidance stage and freezing stage are significantly greater than that in the control stage. The difference is that the LFP power in the avoidance stage is higher than that in the freezing stage. We suspect that the avoidance stage may involve a variety of behaviors and psychology of mice: avoidance, flight, exploration, and freezing. This leads to more neurons participating in it, which increased LFP power. In addition, there was no significant change in neural activity after dropping the same volume of water, as shown in Supplementary Figure S7.

### Responses of Different Neurons in MA to Innate Fear

**Figure 7:**
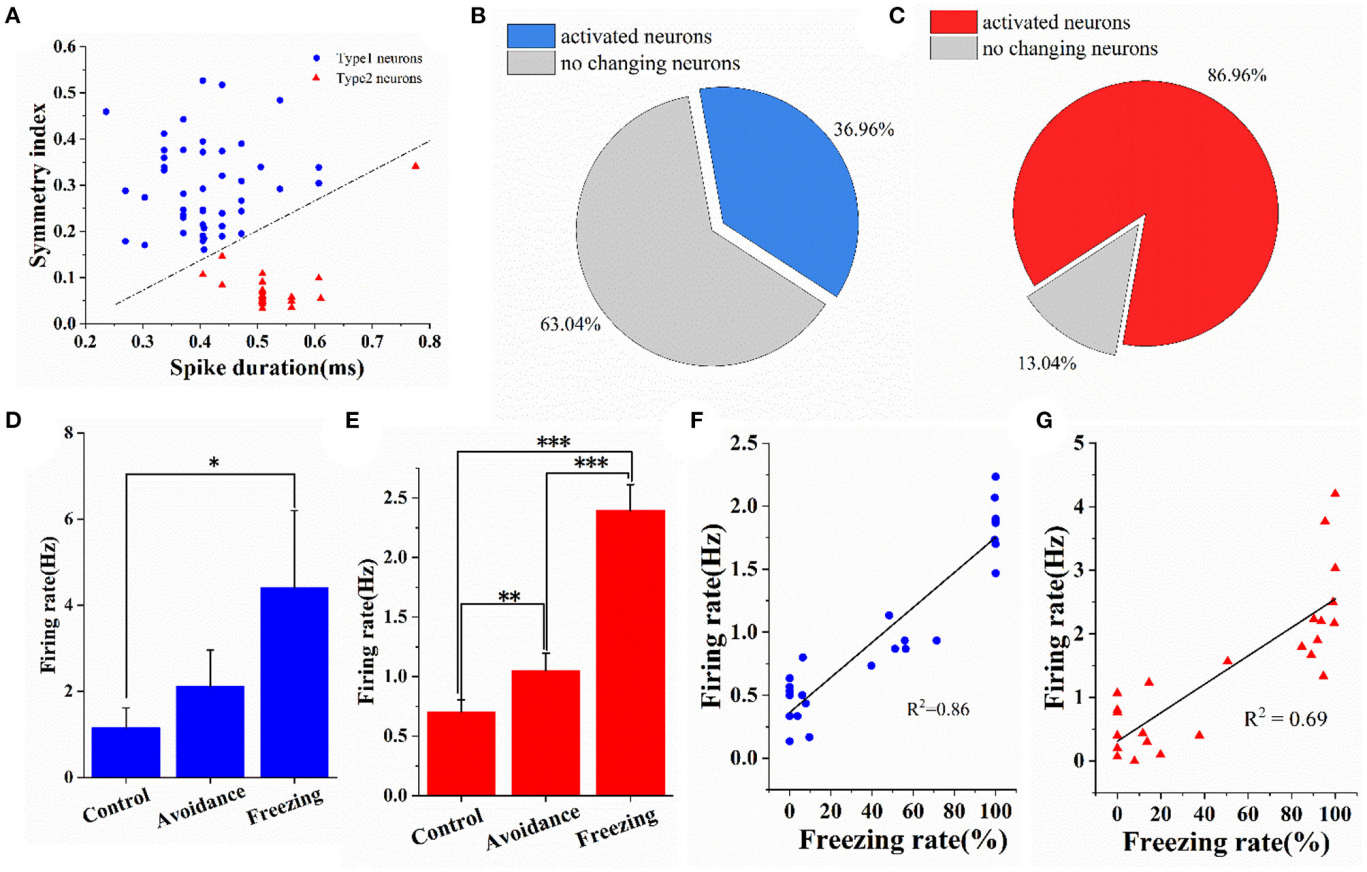
Electrophysiological characteristics of type 1 neurons and type 2 neurons in medial amygdala under fear. **(A)** Classification results of type 1 neurons and type 2 neurons (*n* = 69 neurons). **(B,C)** The proportion of type 1 neurons and type 2 neurons activated under fear. **(D,E)** The firing rate of activated type 1 neurons and type 2 neurons in three stages. **(F,G)** Linear fitting graph of one typical type 1 neuron and one typical type 2 neuron firing rate with freezing rate of mice (bin = 30 s). Data are means ± SE. One-way repeated measures ANOVA. *p < 0.05; **p < 0.01; ***p < 0.001.

We differentiated neurons by waveforms into two classes, namely, type 1 and type 2, according to the principles commonly applied to cortical extracellular recordings. Specifically, we extracted two features of spike waveform, namely, symmetry and pulse width, and combined them with the clustering algorithm to divide the detected neurons into type 1 neurons and type 2 neurons (Barthó et al., 2004; Riera et al., 2014; English et al., 2017) (the parameters for this calculation are shown in Supplementary Figure S8). The classification results are shown in Figure 7A. Among the 69 neurons, there were 46 type 1 neurons and 23 type 2 neurons. Supplementary Figure S9 shows the average waveforms of these 69 neurons. At the same time, we counted the proportion of neurons significantly activated in the freezing stage. Neurons whose firing rate was significantly higher (one-way ANOVA, *p* < 0.05) in the freezing stage than in the control stage were considered activated neurons. The results showed that nearly 87% of type 2 neurons and 37% of type 1 neurons were significantly activated (Figures 7B,C). Then, we calculated the firing rate of activated neurons in three stages. Compared with the control stage, the firing rate of type 1 neurons and type 2 neurons in the freezing stage increased by 283 and 240%, respectively (Figures 7D,E). In addition, we calculated the synchronous changes in the firing rate of different types of neurons and the freezing rate (bin = 30 s) (Supplementary Figure S10). It can be seen that the firing rate of activated neurons has the same trend as the freezing rate of mice (Supplementary Figures S10A,C). We then calculated the correlation between the firing rate of activated neurons and the freezing rate in mice. In addition, we found a good linear correlation between the firing rate of partially activated neurons and the freezing rate. Supplementary Figure S11 shows a linear fitting coefficient between the firing rates of all activated type 1 neurons (*n* = 17) and type 2 neurons (*n* = 20) with the freezing rate of mice. The average value of type 2 neurons fitting coefficient is greater than that of type 1 neurons. But there was no significant difference between them. The correlation coefficient distribution of activated type 1 neurons is relatively scattered, and the correlation coefficient distribution of type 2 neurons is relatively concentrated. The correlation coefficient between the firing rate of the two types of neurons and the freezing rate can reach up to 0.86 and 0.69 (Figures 7F,G).

## Discussion

Electrophysiological detection is an important method of neuroscience research. *In vivo* electrophysiological detection of freely behaving animals is closer to the true physiological state of animals than *in vitro* and under anesthesia, which can also help people better study the neural activities in a certain state. As an important tool for electrophysiological detection, MEAs have great advantages for the *in vivo* detection of freely behaving mice. Electrode surface modification is a necessary means to improve the excellent performance. The combination of metal nanoparticles and conductive polymers can integrate their advantages and further improve the performance of the electrode. Compared with PtNPs-modified electrode, PtNPs/PEDOT:PSS-modified electrode has lower phase delay, larger charge storage capacity, and more stable electrochemical and mechanical properties.

Amygdala is considered to be the emotional center of the brain and plays an important role in the learning and regulation of fear. The main functions of the amygdala subnucleus seem to be separated in different fear types, and CEA is associated with both innate fear and learned fear (Ciocchi et al., 2010; Isosaka et al., 2015). The lateral nucleus (LA) and BLA are mainly involved in learned fear (Ehrlich et al., 2009; Duvarci and Pare, 2014; Janak and Tye, 2015), while MA is mainly involved in olfactory-related innate fear (Bergan et al., 2014; Govic and Paolini, 2015; Silva et al., 2016). At present, there are few studies on the neural coding of MA in freely behaving mice under persistent fear. We used MEAs to study the electrophysiology of mouse MA under 2MT-induced fear to fill the gap in this part.

In the previous electrophysiological studies on predator odor-induced fear, people mainly focused on the neural activities in a short time before and after odor generation (Bergan et al., 2014; Govic and Paolini, 2015; Deng et al., 2016). We think that the process from dropping 2MT to the mouse smelling 2MT and then the mouse confirming that there may be a danger is not instantaneous. In this study, the mouse may go through many processes such as confirming information, selecting defense methods, and executing defense means. So we conducted a longer-term dimension of detection and analysis to more accurately explore the electrophysiological changes in mice's MA under innate fear. In the behavioral experiment, we also found that after adding 2MT, the mice would panic and want to escape, and then exhibit strong freezing. These are all the defensive behaviors of mice. So we divided the entire process into three stages, namely, control, avoidance, and freezing. In the avoidance stage, the mouse has just encountered danger and tried various defense methods, while the freezing stage is more like a state when the danger exists for a long time. Also, we found significant differences in neural activity in the three stages. The average firing rate of neurons in the avoidance stage and freezing stage increased gradually, indicating that the excitability of MA neurons in mice was stronger when the danger existed for a long time. In addition, the LFP power in the avoidance stage is higher than that in the freezing stage, which indicates that more neurons are involved in more types of defense behaviors (escape, jumping, freezing, etc.) in the avoidance stage.

The neurons in MA are mainly glutamatergic neurons and GABAergic neurons (Bombardi, 2014). Glutamatergic neurons and GABAergic neurons in MA received olfactory synaptic input from their upstream accessory olfactory bulb. Most of the MA neurons projected into the hypothalamus are glutamatergic neurons, and their morphology and intrinsic physiological properties are similar to cortical neurons (Bian et al., 2008). Moreover, the hypothalamus-projecting neurons received inhibitory input from the local GABAergic neurons. A recent study showed that a kind of dopamine receptor-expressing neuron projecting to the hypothalamus in MA is mainly glutamatergic, which regulates defense behavior under innate fear (Miller et al., 2019). This makes us interested in the role of glutamatergic and GABAergic neurons in 2MT-induced fear. We tried to classify the detected MA neurons into type 1 neurons and type 2 neurons using the two parameters of symmetry and pulse width. We speculate that type 1 and type 2 neurons may be GABAergic and glutamatergic respectively, that is, putative GABAergic and putative glutamatergic neurons. Because the ratio of these two types of neurons is 2:1 in our results, similar to the results of previous studies (Keshavarzi et al., 2014). The results show that about 87% of putative glutamatergic neurons and 37% of putative GABAergic neurons are significantly activated in the freezing stage (Figures 7B,C), which may indicate that glutamatergic neurons play a more important role in the circuit.

In our experiments, there is no direct evidence for a causal relationship between the increase in firing rates of MA neurons and the increase in freezing rate. Therefore, it cannot be ruled out that the simultaneous increase of MA neuronal firing rate and freezing rate may be due to the fact that the two are regulated by different circuits in parallel, as this will require further experiments to prove. However, considering the previous studies on the regulation of MA, for example, both permanent and temporary MA injuries reduce predator odor-induced freezing (Li et al., 2004; Müller and Fendt, 2006), it can be speculated that some activated neurons in MA are involved in the encoding and transmission of neural information under innate fear from our results. The firing rate of some activated putative GABAergic neurons and glutamatergic neurons and the freezing rate of mice have the same trend under fear, which may also explain the correlation between MA neural activity and freezing behavior. The correlation coefficient of putative GABAergic neurons is relatively scattered (Supplementary Figure S11), which may be due to the diverse functions of GABAergic neurons in the circuit (Bian, 2013; Keshavarzi et al., 2014).

## Conclusion

We fabricated MEAs and used them to perform electrophysiological detection and analysis on MA of freely behaving mice under persistent innate fear. PtNPs/PEDOT:PSS was electroplated on the electrode to improve detection performance. According to animal behavior, we divided the entire behavioral process of mice into three stages, namely, control, avoidance, and freezing. On the whole, the average neuron spike firing rate increased significantly in the latter two stages, and the freezing stage was higher than the avoiding stage. This indicates that some MA neurons continue to be in a state of excitement when the danger persists. The LFP power is mainly concentrated in the δ (1–4 Hz) and θ (4–8 Hz), and the latter two stages also have a significant increase. The detected neurons are divided into putative GABAergic (type 1) and putative glutamatergic (type 2) neurons according to the neurons' waveforms. About 87% of putative glutamatergic neurons and 37% of putative GABAergic neurons are significantly activated after fear. The firing rate of some activated neurons was positively correlated with the freezing rate, which may indicate that MA neurons may be involved in the transmission of neural information encoding fear emotion.

## Data Availability Statement

The original contributions presented in the study are included in the article/Supplementary Material, further inquiries can be directed to the corresponding authors.

## Ethics Statement

The animal study was reviewed and approved by Institutional Animal Care and Use Committee at Aerospace Information Research Institute, Chinese Academy of Science.

## Author Contributions

XC and PF conceived the study and carried out the experiments. PF analyzed the related data and was a major contributor in writing the manuscript. YS and YD interpreted the results. YS, BL, YW, YD, JX, EH, ZX, GY, and FM provided the technical support to this manuscript. YS, YD, JL, and MW provided help in scientific writing. All the authors read and approved the final manuscript.

## Funding

This work was sponsored by the National Key Research and Development Program (2017YFA0205902), the National Natural Science Foundation of China (62121003, 61960206012, 61971400, 62171434, 61975206, and 61973292), and the Scientific Instrument Developing Project of the Chinese Academy of Sciences (GJJSTD20210004).

## Conflict of Interest

The authors declare that the research was conducted in the absence of any commercial or financial relationships that could be construed as a potential conflict of interest.

## Publisher's Note

All claims expressed in this article are solely those of the authors and do not necessarily represent those of their affiliated organizations, or those of the publisher, the editors and the reviewers. Any product that may be evaluated in this article, or claim that may be made by its manufacturer, is not guaranteed or endorsed by the publisher.
