# Supplementary material for: PtNPs/PEDOT:PSS-Modified Microelectrode Arrays Reveal Electrophysiological Activities of Different Neurons in Medial Amygdala of Mice Under Innate Fear
Source: Front Neurosci. 2022 May 10;16:868235. doi: 10.3389/fnins.2022.868235 (PMC9127061; doi:10.3389/fnins.2022.868235)
Supplement: Supplementary file 1 [file Data_Sheet_1.docx]

Supplementary Material


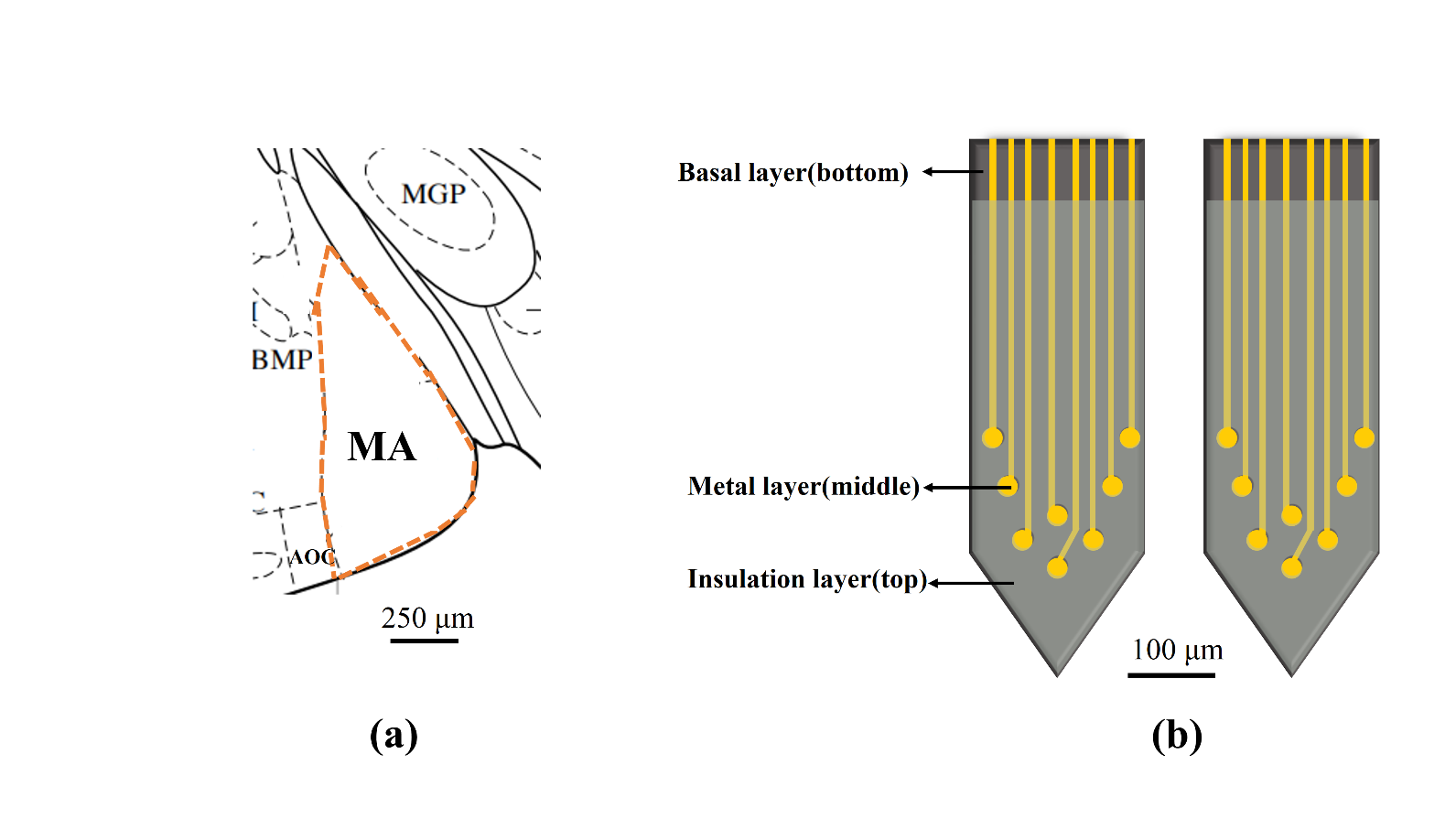


**Supplementary Figure 1.** The Anatomy of the medial amygdala (MA) and schematic diagram of MEA. (a) Anatomy of the brain area of the medial amygdala (MA); (b) Schematic diagram of electrode, which mainly includes three-layer structure. Basal layer: Si. Metal layer: Ti/Pt. Insulation layer: Si_3_N_4_/SiO_2_.


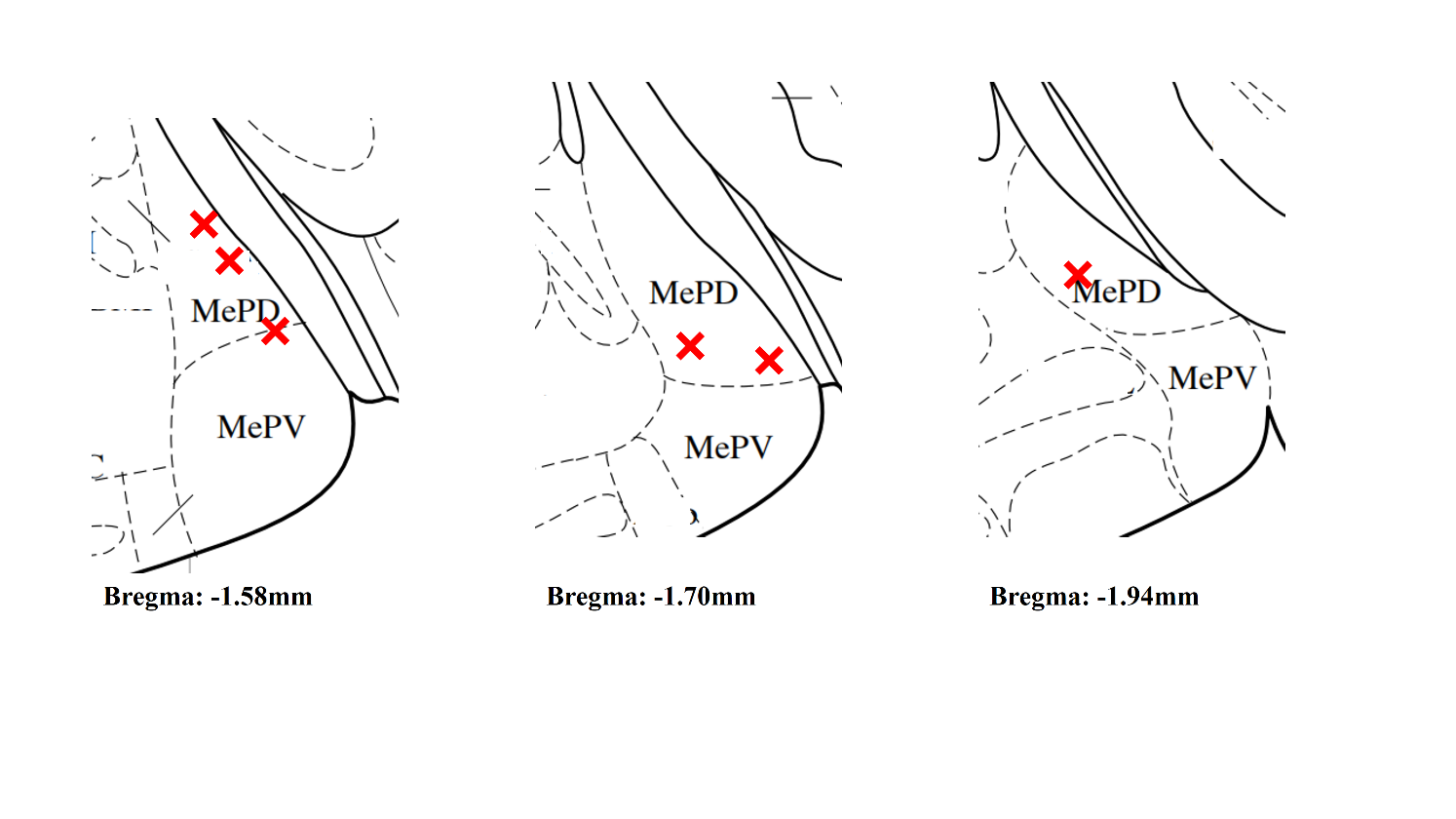


**Supplementary Figure 2.** Schematic maps of the positions of electrodes within the MA in 6 recorded animals.


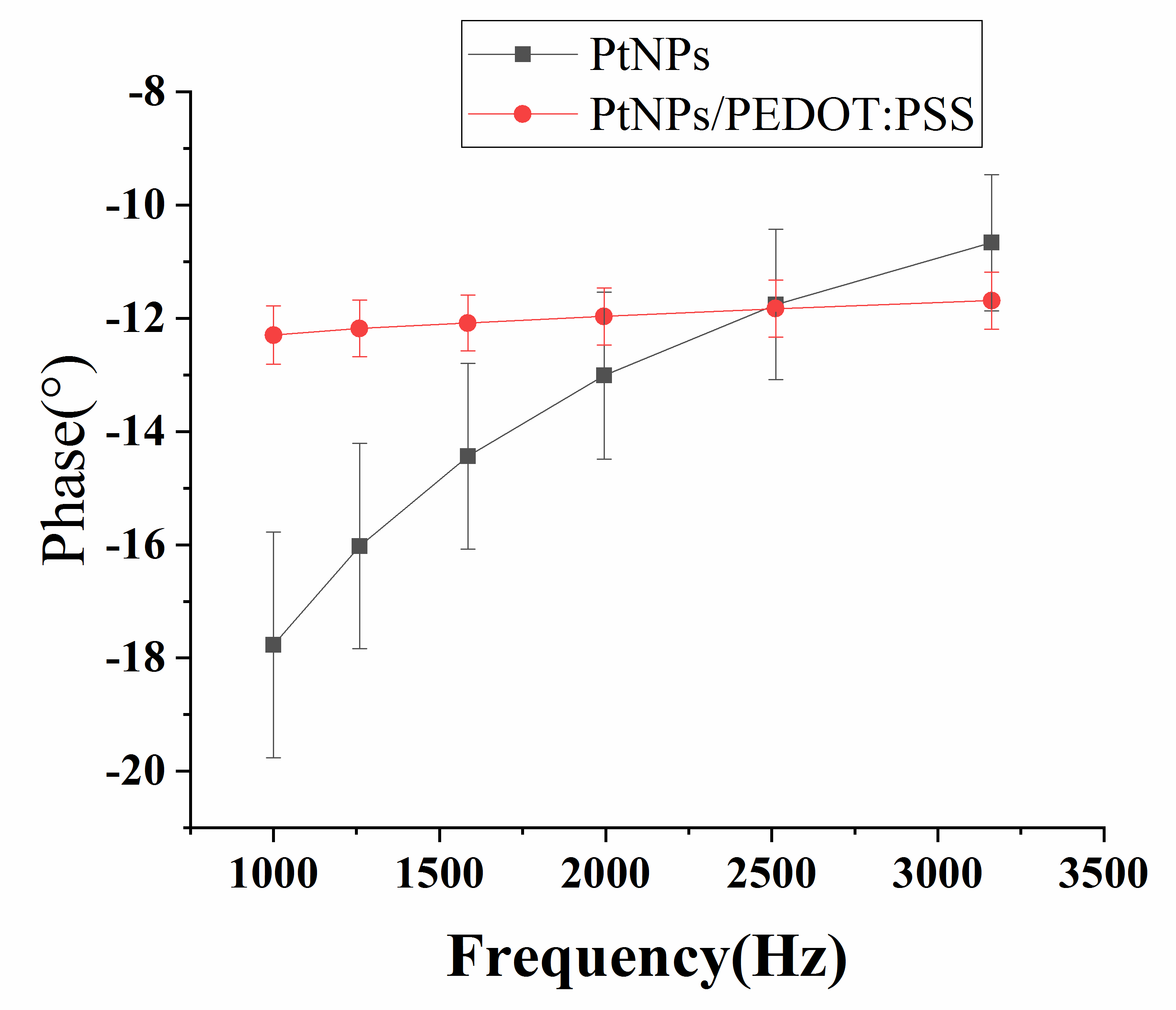


**Supplementary Figure 3**. Average phase delay of PtNPs and PtNPs/PEDOT:PSS modified electrodes at 1-3 kHz (n = 5 recording sites).





**Supplementary Figure 4**. Phase delay (1 kHz) of PtNPs and PtNPs/PEDOT:PSS modified electrode after CV sweeping (6000 circle) and ultrasonic oscillation (50W, 100 min). One way repeated ANOVA, *p < 0.05; **p < 0.01; ***p < 0.001; ns, p > 0.05. n = 4 recording sites.

Table 1

Electrode impedance at 1 kHz under different modifications

| **Modification method** | **mean ± SE (KΩ)** |
| --- | --- |
| Bare | 1507.5 ± 94.1 |
| PEDOT:PSS | 94.0 ± 3.6 |
| PtNPs | 21.0 ± 0.5 |
| PtNPs /PEDOT:PSS | 27.0 ± 2.3 |

n = 5 recording sites.

Table 2

Statistical analysis of impedance at 1 kHz under different modifications

| **Modification method** | **Significance** |
| --- | --- |
| Bare VS PEDOT:PSS | *** |
| Bare VS PtNPs | *** |
| Bare VS PtNPs/PEDOT:PSS | *** |
| PEDOT:PSS VS PtNPs | *** |
| PEDOT:PSS VS PtNPs/PEDOT:PSS | *** |
| PtNPs VS PtNPs/PEDOT:PSS | ns |

One way repeated ANOVA, ***p < 0.001; ns, p > 0.05. n = 5 recording sites.

Table 3

Electrode phase delay at 1 kHz under different modifications

| **Modification method** | **mean ± SE (**°**)** |
| --- | --- |
| Bare | -80.0 ± 2.4 |
| PEDOT:PSS | -45.6 ± 0.2 |
| PtNPs | -17.8 ± 2.0 |
| PtNPs /PEDOT:PSS | -12.3 ± 0.5 |

n = 5 recording sites.

Table 4

Statistical analysis of phase delay at 1 kHz under different modifications

| **Modification method** | **Significance** |
| --- | --- |
| Bare VS PEDOT:PSS | *** |
| Bare VS PtNPs | *** |
| Bare VS PtNPs/PEDOT:PSS | *** |
| PEDOT:PSS VS PtNPs | *** |
| PEDOT:PSS VS PtNPs/PEDOT:PSS | *** |
| PtNPs VS PtNPs/PEDOT:PSS | * |

One way repeated ANOVA, ***p < 0.001; *p < 0.05. n = 5 recording sites.


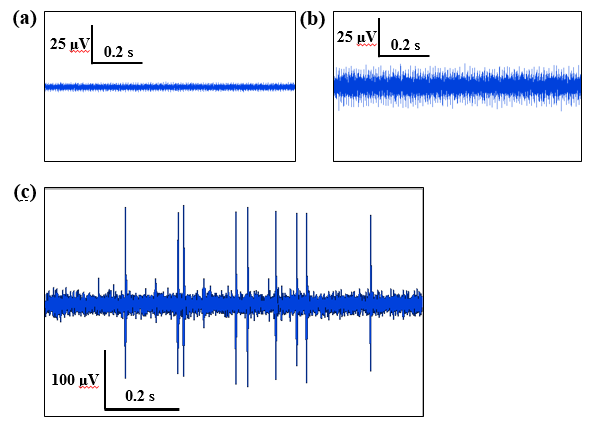


**Supplementary Figure 5**. (a) Typical example of 1s background noise of PtNPs/PEDOT:PSS modified electrode in PBS. (b) Typical example of 1s background noise of PtNPs modified electrode in PBS. (c) Typical example of neural signal acquired from PtNPs/PEDOT:PSS modified electrode.


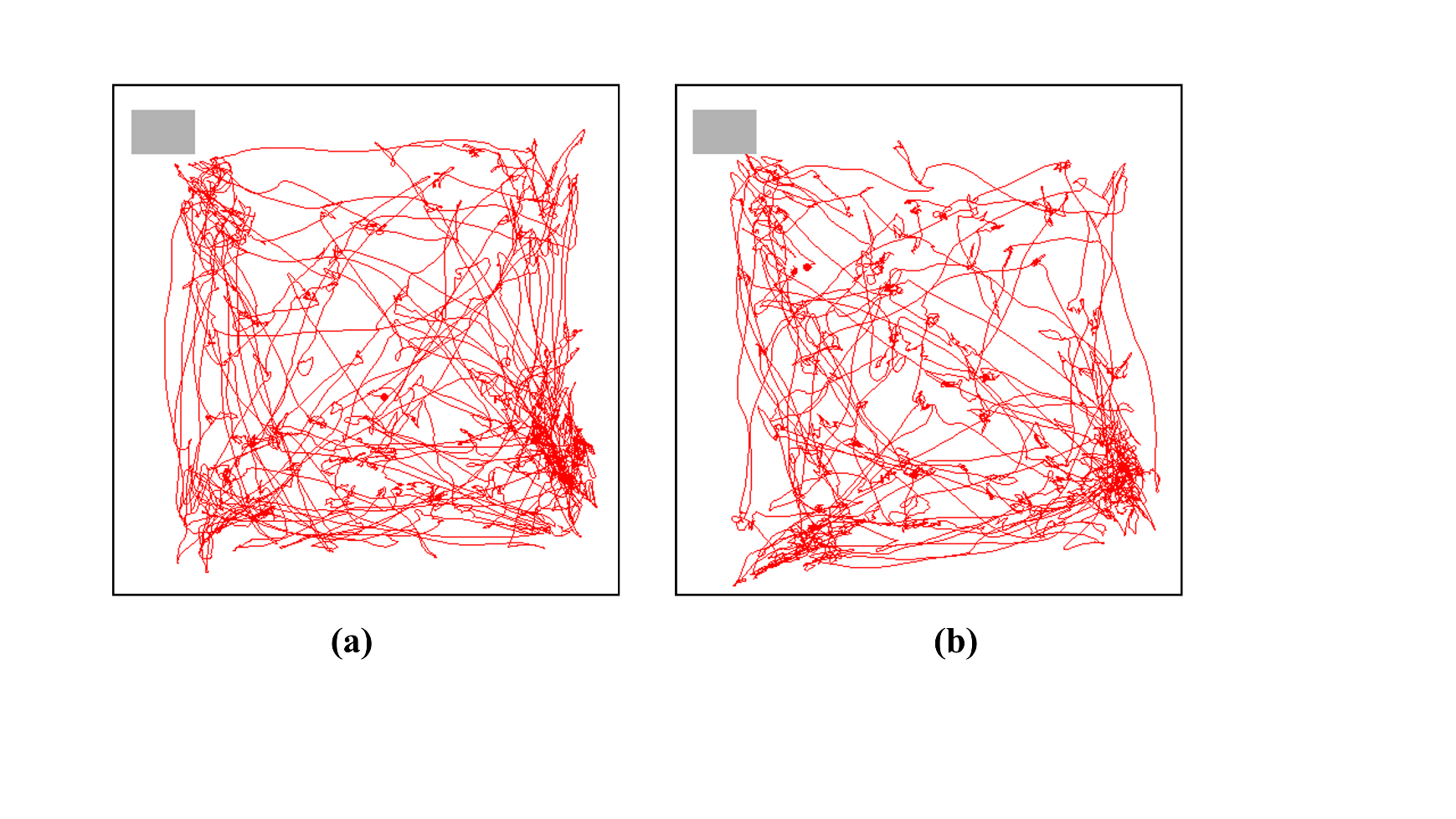


**Supplementary Figure 6**. Trajectory diagram of the mouse in the control state and after dripping water. (a)Trajectory diagram of the mouse in the control state (5 min); (b) Trajectory diagram of the mouse after dripping water (5 min). The gray area represents the 2MT paper range.


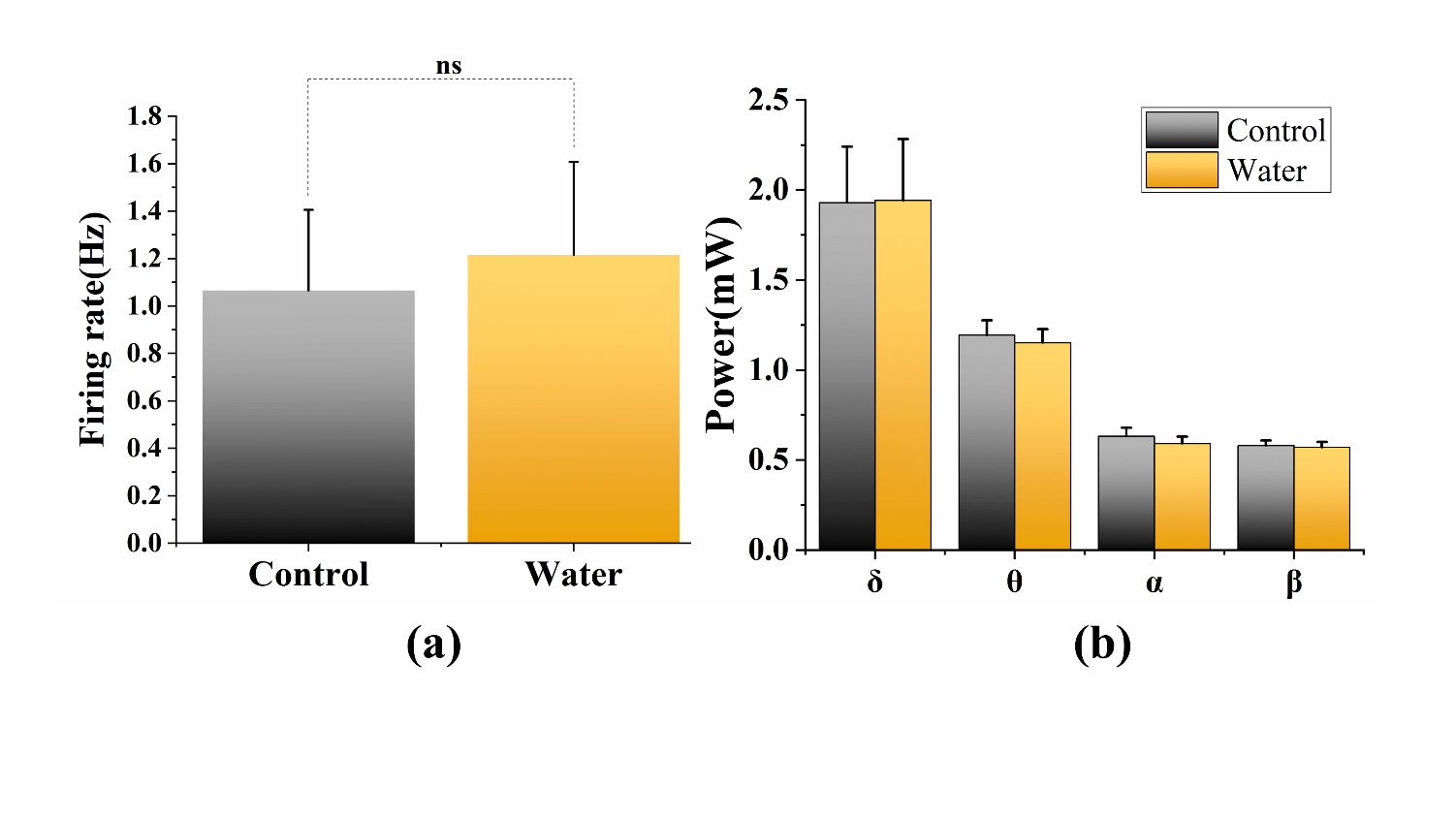


**Supplementary Figure 7**. Neural activity of mouse MA in control state and after adding water. (a) Spike firing rate of control group and water group; (b) LFP power of different frequency bands in control state and after adding water. Data are means ± SD. One-way ANOVA. ns, p > 0.05. n = 3 mice.


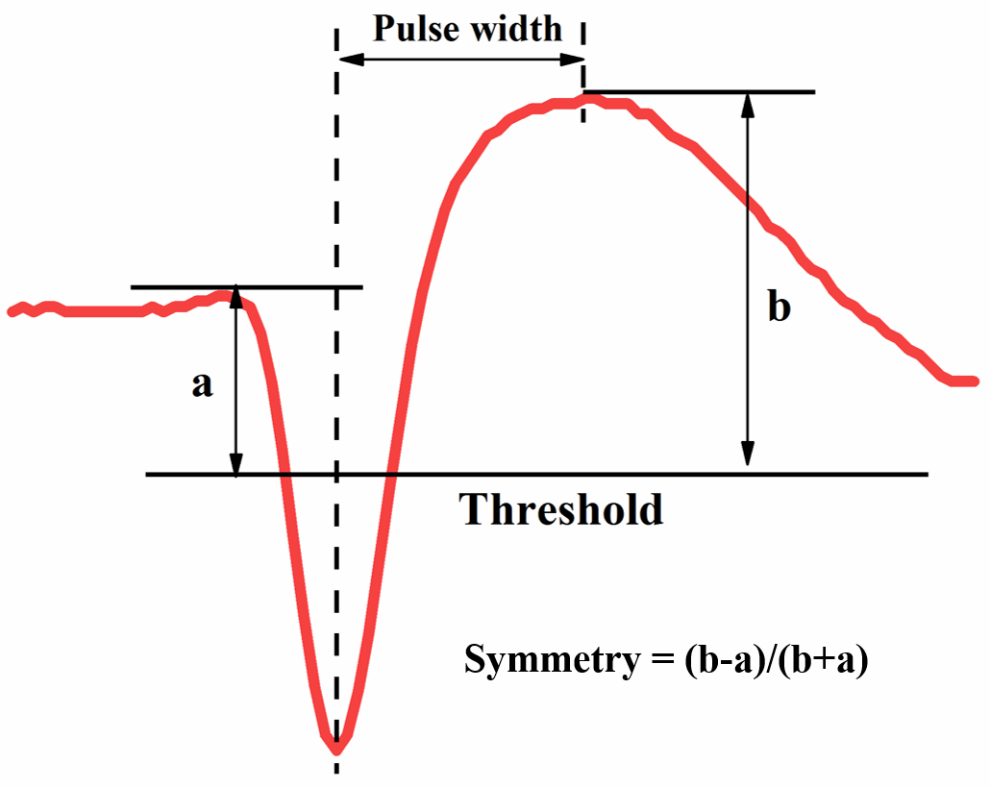


**Supplementary Figure 8**. Schematic diagram of symmetry and pulse width.

(a)
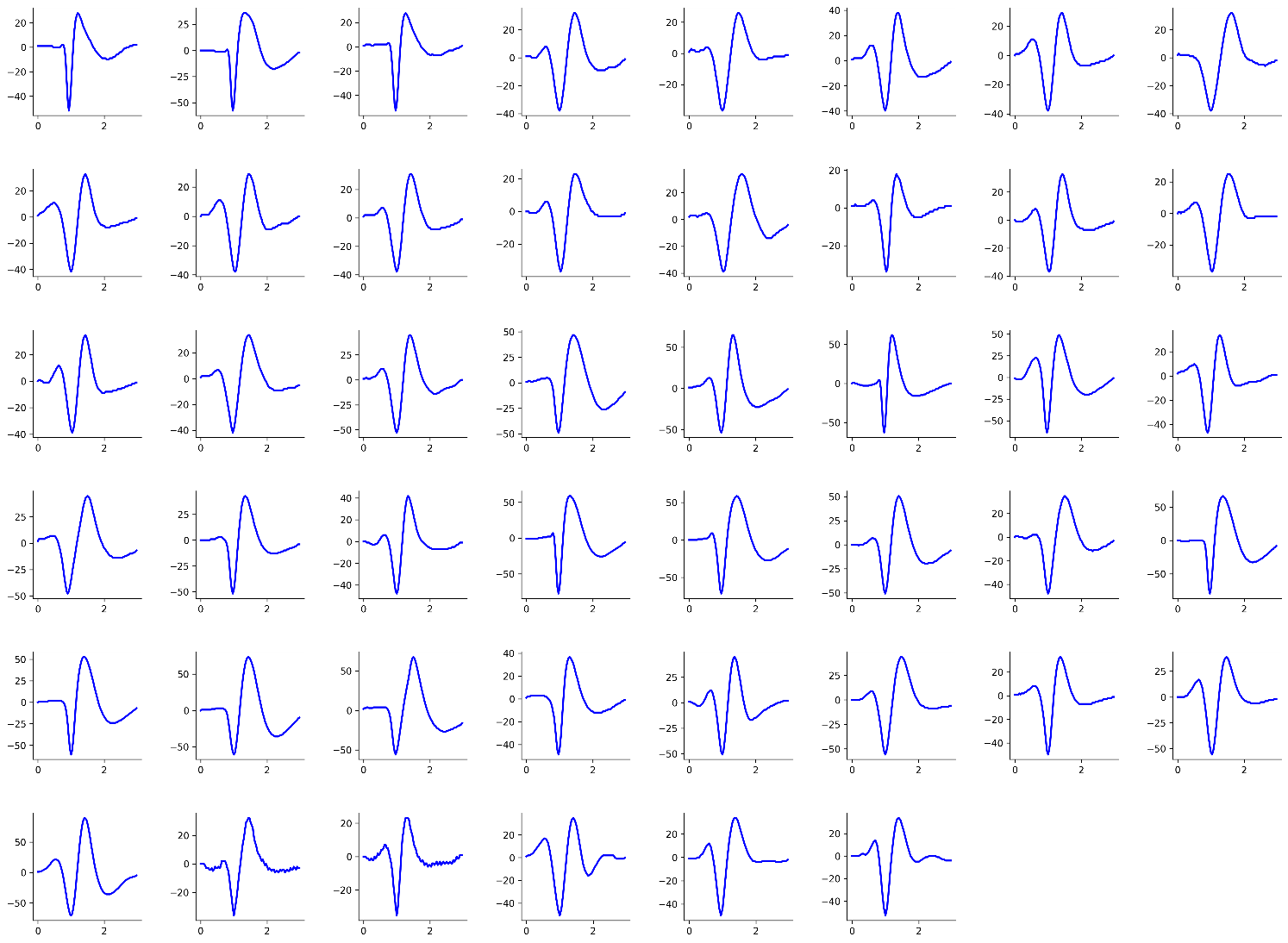


(b)


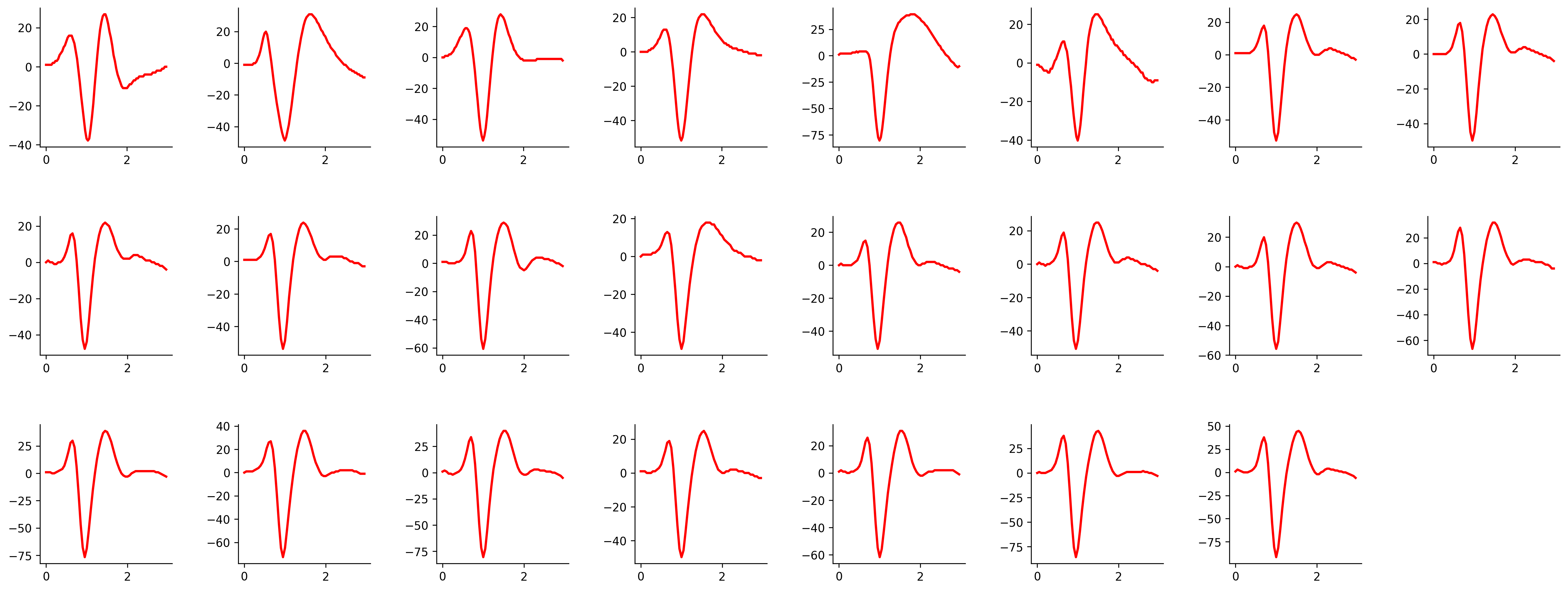


**Supplementary Figure 9**. (a) Average waveforms of 46 type 1 neurons. (b) Average waveforms of 23 type 2 neurons. The unit of abscissa is ms, and the unit of ordinate is μV.


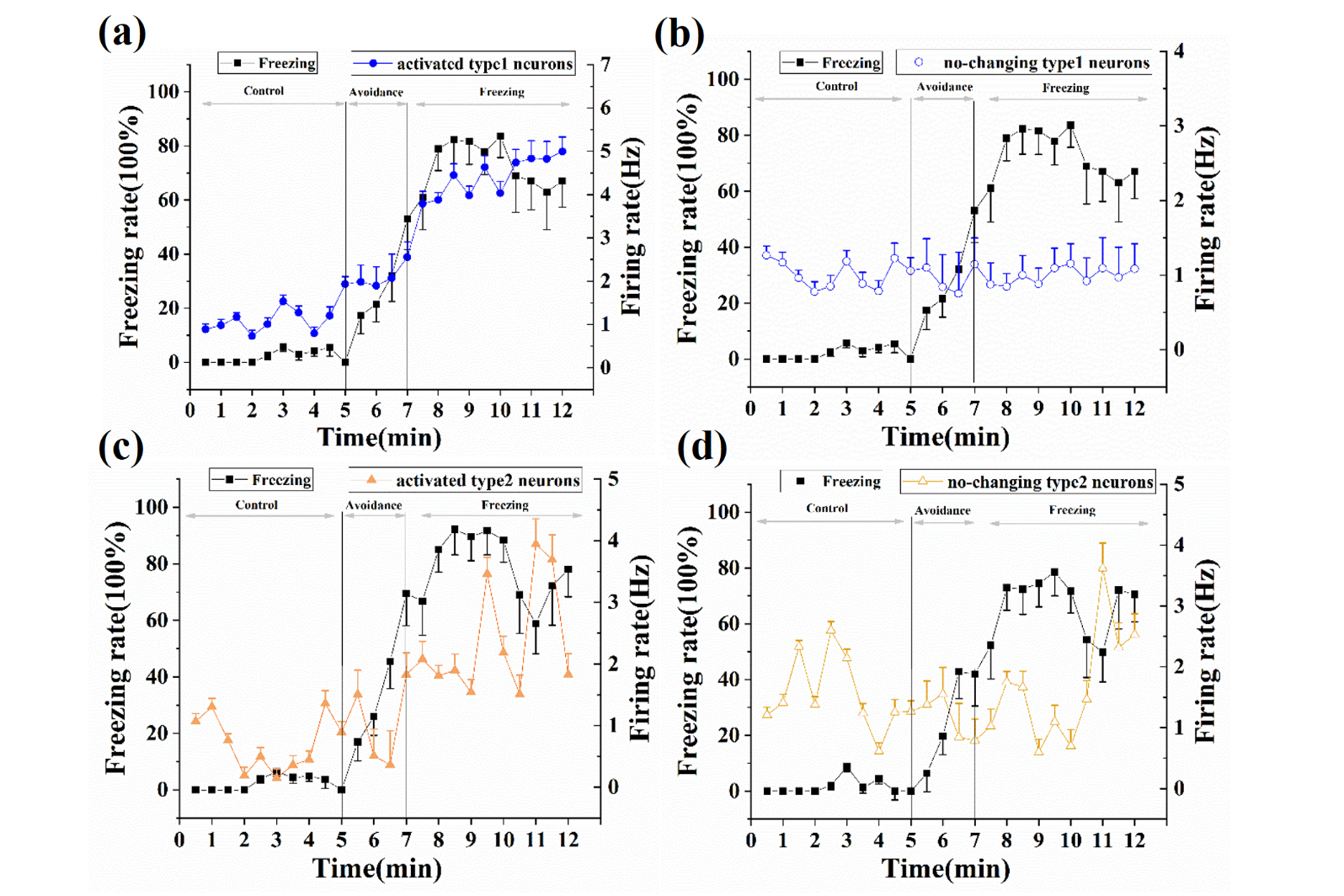


**Supplementary Figure 10**. (a) Synchronous changes of firing rate of activated type1 neurons and freezing rate of mice (mice = 6, neurons = 17). (b) Synchronous changes of firing rate of no-changing type1 neurons and freezing rate of mice (mice = 6, neurons = 29). (c) Synchronous changes of firing rate of activated type2 neurons and freezing rate of mice (mice = 4, neurons = 20). (b) Synchronous changes of firing rate of no-changing type2 neurons and freezing rate of mice (mice = 3, neurons = 3). Bin = 30s.

**

**

**Supplementary Figure 11**. Linear fitting coefficient between firing rates of activated type1 neurons (n=17 neurons, blue) and activated type2 neurons (n=20 neurons, red) and freezing rate of mice.
